# Supplementary material for: Parameter-free representations outperform single-cell foundation models on downstream benchmarks
Source: bioRxiv. 2026 Feb 13:2026.02.11.705358. Preprint. [Version 1] doi: 10.64898/2026.02.11.705358 (PMC12918925; doi:10.64898/2026.02.11.705358)
Supplement: Supplement 1 [file NIHPP2026.02.11.705358v1-supplement-1.pdf]

bioRxiv preprint doi: https://doi.org/10.64898/2026.02.11.705358; this version posted February 13, 2026. The copyright holder for this preprint (which was not certified by peer review) is the author/funder, who has granted bioRxiv a license to display the preprint in perpetuity. It is made available under aCC-BY 4.0 International license.

Appendix A: Materials and Methods

1. scTOP package

For cross-species annotation, we used our previously published method, single-cell Type Order Parameter (scTOP) [31]. Moreover, the pre-processing function of scTOP was applied to every task in our work. scTOP quantifies cellular identity by projecting a cell's gene expression profile onto a defined cell type space. Each cell is processed independently to minimize batch effects.

This preprocessing calculation is performed as follows. First, each cell's raw counts, x_j, are normalized by the total counts in that cell (for $j = 1, \ldots, G$):

$$x_j^{\text{norm}} = \frac{x_j}{\sum_{k=1}^{G} x_k}$$

This normalized value is then transformed using a $\log_2(x+1)$ function:

$$y_j = \log_2(x_j^{\text{norm}} + 1).$$

The y_j value is ranked relative to all other transformed gene values in that cell, yielding R_j. The rank is converted to a percentile rank, p_j, using the convention:

$$p_j = \frac{R_j}{G+1}$$

This percentile is then converted to a z-score, S_j, using the inverse of the standard normal cumulative distribution function (CDF), Φ^{-1}:

$$S_j = \Phi^{-1}(p_j)$$

The resulting G-dimensional vector, composed of the S_j values, is used for the projection. Therefore, S_j is the preprocessed single cell gene expression vector.

Next, a reference basis is constructed from existing single-cell atlases to define the C cell types of interest. For each of the C cell types (e.g., Fibroblast, Macrophage, Basal), the gene expression profiles from a representative population (typically 100-200 cells) are first normalized individually (by total counts per cell, as above), and then averaged to create a single archetypal vector. This vector is then processed using the exact same $\log_2$ transformation, ranking, and z-scoring procedure (applied to the single averaged vector) to create the final reference vector, ξ_i^μ, with $\mu = (1, ..., C)$. These C vectors form a non-orthogonal basis for the C-dimensional cell type space.

The final scTOP scores, a^μ, are calculated by projecting the query cell's vector (composed of S_j values) onto this non-orthogonal basis. This is not a simple dot product but a de-correlated projection that accounts for the similarity between the reference types. First, an overlap matrix, $A_{\mu\nu}$, is

bioRxiv preprint doi: https://doi.org/10.64898/2026.02.11.705358; this version posted February 13, 2026. The copyright holder for this preprint (which was not certified by peer review) is the author/funder, who has granted bioRxiv a license to display the preprint in perpetuity. It is made available under aCC-BY 4.0 International license.

computed, where each element is the dot product of two reference cell type vectors:

$$A_{\mu\nu} = \sum_{j=1}^{G} \xi_j^{\mu} \xi_j^{v}.$$

The final score, a^{μ}, for the query cell's similarity to cell type μ is a sum of the cell's similarity to all reference types, weighted by this inverse matrix:

$$a^{\mu} = \sum_{v=1}^{C} \sum_{j=1}^{G} [A_{\mu\nu}]^{-1} \xi_j^{v} S_j.$$

The result is a set of C scores for the query cell, representing its coordinates in the de-correlated cell type space. We then annotate the cell as the cell type with highest projection.

2. ANOVA gene selection

For human cell annotation and disease state identification, after preprocessing samples using scTOP, we computed ANOVA F-statistic on the dataset, in order to select informative genes for each tissue. Let $x_{i,g}$ denote the standardized expression of gene g in cell i, and let the dataset contain C cell types with class means $\bar{x}_g^{(c)}$ and overall mean $\bar{x}_g$. The between-class variance is

$$S_B(g) = \sum_{\mu=1}^{C} n_{\mu} \left(\bar{x}_g^{(\mu)} - \bar{x}_g\right)^2,$$

and the within-class variance is

$$S_W(g) = \sum_{\mu=1}^{C} \sum_{i \in C_{\mu}} \left(x_{i,g} - \bar{x}_g^{(\mu)}\right)^2.$$

The ANOVA score is

$$F(g) = \frac{S_B(g)/(C-1)}{S_W(g)/(N-C)}.$$

The N_{ANOVA} genes with the highest values of $F(g)$ were retained. This is computed in the training set. The selected genes during training are applied to the test set.

bioRxiv preprint doi: https://doi.org/10.64898/2026.02.11.705358; this version posted February 13, 2026. The copyright holder for this preprint (which was not certified by peer review) is the author/funder, who has granted bioRxiv a license to display the preprint in perpetuity. It is made available under aCC-BY 4.0 International license.

3. Standardization

After ANOVA selection, the genes in a cell are no longer necessarily normalized. For this reason, for each cell i, we standardize the gene expression as follows:

$$S_i' = \frac{S_i - \mu_i}{\sigma_i},$$

where S_i represent the gene expression vector for cell i after ANOVA, μ_i is the mean of this vector, and σ_i its standard deviation.

4. Principal Component Analysis

After ANOVA selection and standardization, Principal Component Analysis (PCA) is applied. PCA reduces the dimensionality of the data by identifying the primary axes of variance, which serves to capture the most significant biological signals while filtering out stochastic noise.

Let $\mathcal{S}'$ denote the $N \times G'$ matrix of standardized, gene-selected values, where N is the number of cells and G' is the number of retained genes. The sample covariance matrix is

$$\mathbf{\Sigma} = \frac{1}{N-1}\mathcal{S}'^{\top}\mathcal{S}'.$$

PCA solves the eigenvalue problem to find the principal directions (eigenvectors) $\mathbf{u}_j$:

$$\mathbf{\Sigma}\mathbf{u}_j = \lambda_j \mathbf{u}_j,$$

where λ_j is the eigenvalue representing the variance captured by $\mathbf{u}_j$. The principal directions are ordered such that $\lambda_1 \geq \lambda_2 \geq \cdots \geq \lambda_{G'}$. By projecting a cell's standardized vector $\mathbf{s}_i'$ (a column vector representing the i-th cell's data from $\mathcal{S}'$) onto the first d principal directions, we create a new, low-dimensional representation $\mathbf{z}_i$:

$$\mathbf{z}_i = \mathbf{U}_d^{\top}\mathbf{s}_i', \quad \text{where} \quad \mathbf{U}_d = (\mathbf{u}_1, \ldots, \mathbf{u}_d).$$

This projection retains the majority of the structured variance (captured by the components with large eigenvalues) and discards the higher-order components (with small eigenvalues), which are more likely to represent noise. The resulting vector $\mathbf{z}_i$ is the final, noise-reduced representation of the cell.

bioRxiv preprint doi: https://doi.org/10.64898/2026.02.11.705358; this version posted February 13, 2026. The copyright holder for this preprint (which was not certified by peer review) is the author/funder, who has granted bioRxiv a license to display the preprint in perpetuity. It is made available under aCC-BY 4.0 International license.

5. Logistic regression

For binary classification tasks (e.g., COVID-19 identification), we model the probability that cell i (represented by its PCA vector $\mathbf{z}_i$) belongs to class $y_i = 1$ as

$$P(y_i = 1 \mid \mathbf{z}_i) = \sigma(\mathbf{w}^\top \mathbf{z}_i + b),$$

where $\sigma(t) = 1/(1 + e^{-t})$ is the logistic sigmoid. The parameters $(\mathbf{w}, b)$ minimize the cross-entropy loss

$$\mathcal{L} = -\sum_{i=1}^{N} \left[y_i \log \sigma(\mathbf{w}^\top \mathbf{z}_i + b) + (1 - y_i) \log\bigl(1 - \sigma(\mathbf{w}^\top \mathbf{z}_i + b)\bigr) \right].$$

For multi-class tasks with K types, we use multinomial logistic regression. The probability that a cell belongs to class k is

$$P(y_i = k \mid \mathbf{z}_i) = \frac{\exp(\mathbf{w}_k^\top \mathbf{z}_i + b_k)}{\sum_{\ell=1}^{K} \exp(\mathbf{w}_\ell^\top \mathbf{z}_i + b_\ell)},$$

and the loss is

$$\mathcal{L} = -\sum_{i=1}^{N} \sum_{k=1}^{K} \mathbf{1}(y_i = k) \log P(y_i = k \mid \mathbf{z}_i).$$

(Note: $\mathbf{1}(y_i = k)$ is an indicator function, and k_i in the original prompt is represented by $y_i = k$).

6. Leiden clustering

For the COVID-19 analysis, clustering was performed on the PCA-transformed data ($\mathbf{z}_i$ vectors). The Leiden algorithm maximizes the modularity

$$Q = \frac{1}{2m} \sum_{i,j} \left(A_{ij} - \frac{k_i k_j}{2m} \right) \delta(c_i, c_j),$$

where A_{ij} is the adjacency matrix of the K-nearest-neighbor graph in PCA space, k_i is the degree of node i, $m = \frac{1}{2} \sum_i k_i$, and c_i denotes the cluster assignment of cell i. The algorithm iteratively maximizes Q until convergence.

Each cluster C receives its own logistic regression model trained only on cells within C. A test cell is projected into PCA space, assigned to its nearest Leiden cluster, and evaluated by the corresponding classifier.

bioRxiv preprint doi: https://doi.org/10.64898/2026.02.11.705358; this version posted February 13, 2026. The copyright holder for this preprint (which was not certified by peer review) is the author/funder, who has granted bioRxiv a license to display the preprint in perpetuity. It is made available under aCC-BY 4.0 International license.

7. Cosine similarity analysis

Cosine similarity between average profiles of two groups A and B is computed on the vectors after the preprocessing step from scTOP (before ANOVA or PCA) as

$$\cos(A, B) = \frac{\mu_A \cdot \mu_B}{\|\mu_A\| \, \|\mu_B\|},$$

where μ_A and μ_B are the mean vectors of the groups, using the full-dimensional scTOP-preprocessed vectors $\mathbf{S}_i$:

$$\mu_A = \frac{1}{|A|} \sum_{i \in A} \mathbf{S}_i, \qquad \mu_B = \frac{1}{|B|} \sum_{i \in B} \mathbf{S}_i.$$

(Here $\mathbf{S}_i$ is the G-dimensional vector $(S_{i,1}, \dots, S_{i,G})^\top$ for cell i.)

8. Macro F1-score

For each class c, let precision and recall be

$$\text{Prec}_k = \frac{TP_k}{TP_k + FP_k}, \qquad \text{Rec}_k = \frac{TP_k}{TP_k + FN_k},$$

where TP_k, FP_k, and FN_k denote true positives, false positives, and false negatives, respectively. The F1-score for class k is

$$F1_k = \frac{2 \, \text{Prec}_k \, \text{Rec}_k}{\text{Prec}_k + \text{Rec}_k}.$$

The macro F1-score averages across classes:

$$\text{MacroF1} = \frac{1}{K} \sum_{k=1}^{K} F1_k.$$

9. Hyperparameters choice

To choose the hyperparameter necessary for the classification task on the Tabula Sapiens dataset, we ran a grid search across different values of selected genes for ANOVA selection and number of PCA dimensions. For each choice, we ran a 3-fold cross-validation. The results are shown in Fig 9. As we can see, our results are robust across all choices. Then, we chose 20000 genes and 220 principal components as a way of keeping the maximum information without sacrifice performance.

bioRxiv preprint doi: https://doi.org/10.64898/2026.02.11.705358; this version posted February 13, 2026. The copyright holder for this preprint (which was not certified by peer review) is the author/funder, who has granted bioRxiv a license to display the preprint in perpetuity. It is made available under aCC-BY 4.0 International license.

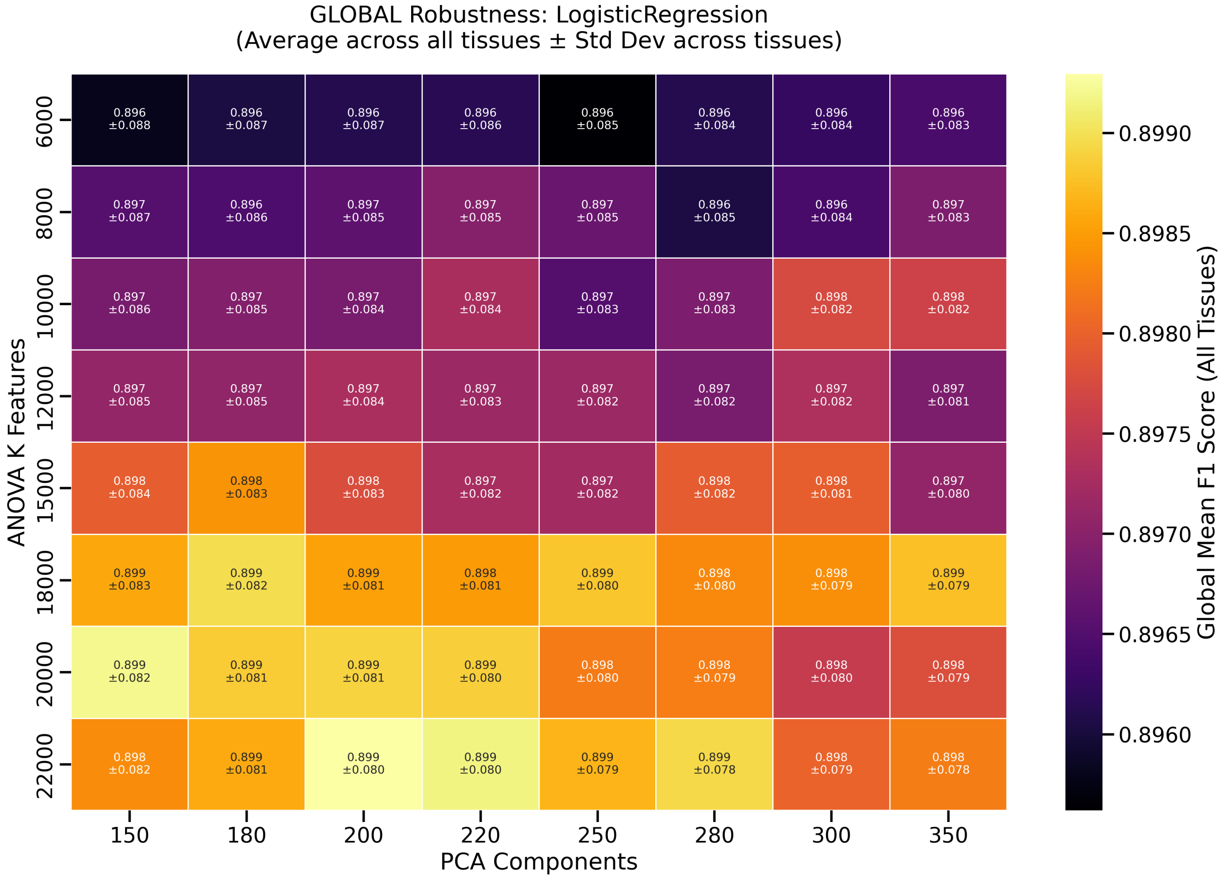

FIG. 9: Macro F1-scores for each choice of number of ANOVA selected genes and number of principal components. We notice that most choices lead to similar results.

10. Gene–gene interaction analysis

All gene–gene interaction analyses were performed on scTOP-processed single-cell expression matrices, as described above. Let $\mathcal{S} \in \mathbb{R}^{N \times G}$ denote the processed expression matrix, where G is the number of genes, N the number of cells, and s_{ig} the processed expression value of gene g in cell i.

For a given transcription factor (TF) t, TF activity was defined directly from its processed expression values. Cells were ranked according to s_{it}, and those in the top quantile q were designated as *TF-active*. Formally, letting $Q_q(s_t)$ denote the empirical q-th quantile of the TF expression distribution, the set of TF-active cells was defined as

$$\mathcal{A}_t = \{i \in \{1, \dots, N\} \mid s_{ti} > Q_q(s_t)\} \,. \tag{A1}$$

bioRxiv preprint doi: https://doi.org/10.64898/2026.02.11.705358; this version posted February 13, 2026. The copyright holder for this preprint (which was not certified by peer review) is the author/funder, who has granted bioRxiv a license to display the preprint in perpetuity. It is made available under aCC-BY 4.0 International license.

This procedure yields a binary conditioning variable indicating whether a cell exhibits high TF activity.

To focus on co-occurrence structure rather than expression magnitude, each gene was modeled as a binary random variable indicating whether it is expressed in a given cell. Specifically, for each gene g and cell i, we defined

$$Y_{ig} = \begin{cases} 1, & \text{if } s_{ig} > 0, \\ 0, & \text{otherwise.} \end{cases} \tag{A2}$$

This binarization step reduces sensitivity to technical variability and mirrors gene-selection strategies employed in recent large-scale transcriptomic models.

Genes expressed in fewer than a fixed fraction of cells (typically 1%) were excluded from downstream analysis to avoid unstable estimates arising from extremely rare events.

For each remaining gene g, we estimated its marginal probability of expression across all cells,

$$\hat{P}(g) = \frac{1}{N} \sum_{i=1}^{N} Y_{ig}, \tag{A3}$$

as well as its conditional probability of expression given TF activity,

$$\hat{P}(g \mid t) = \frac{1}{|\mathcal{A}_t|} \sum_{i \in \mathcal{A}_t} Y_{ig}. \tag{A4}$$

Intuitively, $\hat{P}(g)$ measures how frequently a gene is expressed overall, whereas $\hat{P}(g \mid t)$ measures how frequently it is expressed specifically in TF-active cells.

Gene–TF interactions were quantified using a pointwise mutual information (PMI)–like score,

$$\mathrm{PMI}(g, t) = \log \frac{\hat{P}(g \mid t)}{\hat{P}(g)}. \tag{A5}$$

Positive values indicate genes that are enriched in TF-active cells relative to their baseline expression frequency, while negative values indicate depletion. A small regularization constant was added to both probabilities to avoid numerical instabilities when probabilities approach zero.

For each TF, PMI scores were standardized across genes by converting them to Z-scores,

$$Z_g = \frac{\mathrm{PMI}(g, t) - \mu_t}{\sigma_t}, \tag{A6}$$

where μ_t and σ_t denote the mean and standard deviation of PMI scores for TF t across all analyzed genes. This normalization assumes that most genes are not specifically associated with the TF and therefore provides a natural background distribution.

bioRxiv preprint doi: https://doi.org/10.64898/2026.02.11.705358; this version posted February 13, 2026. The copyright holder for this preprint (which was not certified by peer review) is the author/funder, who has granted bioRxiv a license to display the preprint in perpetuity. It is made available under aCC-BY 4.0 International license.

Two-sided p-values were computed assuming a standard normal distribution, and multiple testing correction was performed using the Benjamini–Hochberg procedure. Genes with false discovery rate–adjusted q-values below 0.05 and positive Z-scores were retained as significant TF-associated genes. In this, the quantile where chose to maximize the genes with q-values below 0.05.

To assess biological relevance, predicted TF–gene associations were compared against independently curated protein–protein interaction data from the STRING database [49]. Interactions supported by STRING were considered validated, providing an external benchmark that is independent of the single-cell expression data.

11. Isomap and Euclidean–Geodesic Distance Analysis

To characterize the geometric structure of the transcriptional data manifold, we compared linear and non-linear notions of distance using principal component analysis (PCA) and Isomap. PCA provides a linear approximation that preserves directions of maximal variance, whereas Isomap estimates distances along the manifold itself by approximating geodesic distances between samples.

Let $\mathcal{S} \in \mathbb{R}^{N \times G}$ denote the set of single-cell gene expression profiles after preprocessing. Isomap begins by constructing a k-nearest-neighbor graph $G = (V, E)$ on these points using Euclidean distances. Two samples S_i and S_j are connected by an edge if S_j is among the k nearest neighbors of S_i, with edge weight

$$w_{ij} = \|S_i - S_j\|_2.$$

This graph provides a local approximation to the underlying manifold.

Geodesic distances on the manifold are then approximated by shortest-path distances on the graph. Specifically, for any pair of samples (i, j), the geodesic distance $d_G(i, j)$ is defined as the minimal sum of edge weights along any path connecting i and j in G. This yields a distance matrix that reflects intrinsic manifold geometry rather than direct Euclidean separation.

To obtain a low-dimensional representation, classical multidimensional scaling (MDS) is applied to the squared geodesic distance matrix D_G^2. MDS finds coordinates $\{y_i\} \subset \mathbb{R}^d$ that minimize the stress function

$$\sum_{i,j} \left(d_G(i,j)^2 - \|y_i - y_j\|_2^2 \right)^2,$$

yielding an embedding that best preserves geodesic distances in a least-squares sense.

To quantify the extent to which non-linear geometry alters global relationships between cells, we compared pairwise Euclidean distances

$$d_E(i,j) = \|S_i - S_j\|_2$$

with the corresponding geodesic distances $d_G(i, j)$. We computed the Pearson correlation coefficient

$$\rho = \operatorname{corr}\big(d_E(i,j),\, d_G(i,j)\big)$$

bioRxiv preprint doi: https://doi.org/10.64898/2026.02.11.705358; this version posted February 13, 2026. The copyright holder for this preprint (which was not certified by peer review) is the author/funder, who has granted bioRxiv a license to display the preprint in perpetuity. It is made available under aCC-BY 4.0 International license.

across all sample pairs. Values of ρ close to one indicate that Euclidean distances already approximate manifold geodesics, implying weak global curvature. Lower values signal stronger non-linear distortion.

Appendix B: Supplemental Information

1. Worst cell types on our pipeline

Similarly to what is reported on [27], we observe that the worst cell types across all tissues are in general T-cells. In Fig. 10 we show the worst 20 cell types F1-scores.

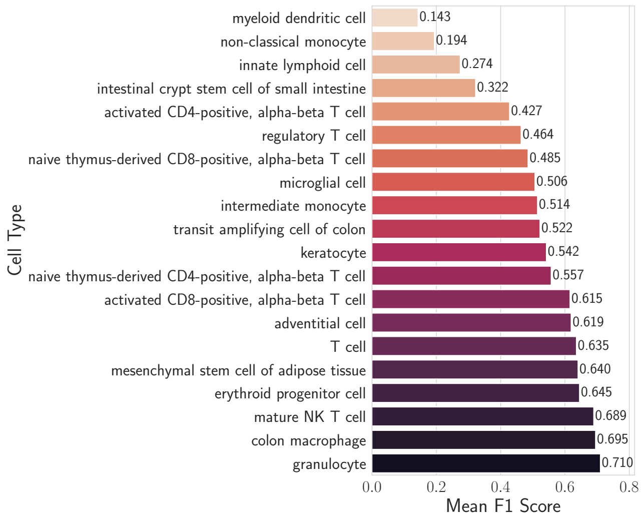

FIG. 10: Worst F1-scores per cell types.

2. Using scTOP only for Tabula Sapiens 2.0

In the main text (Sec. IV), we introduced a pipeline that doesn't use deep learning for annotating human cell types in Tabula Sapiens 2.0 that augments scTOP preprocessing with ANOVA-based gene selection, PCA, and linear classification. Here, we isolate the performance of scTOP alone in order to clarify both its strengths and its limitations on this task, and to explicitly motivate the denoising steps introduced in the main pipeline.

scTOP represents each cell type by a single normalized pseudo-bulk expression vector and classifies individual cells by linear projection onto this reference basis. As shown throughout the main text, this representation is highly effective when the relevant biological structure is low-dimensional and

bioRxiv preprint doi: https://doi.org/10.64898/2026.02.11.705358; this version posted February 13, 2026. The copyright holder for this preprint (which was not certified by peer review) is the author/funder, who has granted bioRxiv a license to display the preprint in perpetuity. It is made available under aCC-BY 4.0 International license.

robust, such as in cross-species annotation or developmental ordering. However, Tabula Sapiens 2.0 poses a fundamentally different challenge: each tissue contains a large number of closely related cell types, scRNA-seq measurements exhibit substantial technical noise and dropout, and within–cell-type transcriptional variability is often comparable to between–cell-type differences. In this regime, collapsing each cell type to a single mean vector discards discriminative information that is essential for fine-grained classification.

Figure 11A shows the macro F1-score obtained by scTOP alone across tissues, evaluated using the same train–test splits and metrics as in the main text and prior foundation-model benchmarks. While scTOP performs well above chance and captures coarse cell-type structure, it consistently underperforms both foundation-model embeddings and the scTOP+ANOVA pipeline. This performance gap reflects a characteristic failure mode of centroid-based representations in noisy, high-dimensional settings: projections onto mean vectors are dominated by stochastic variability rather than cell-type–specific signal.

The improvement obtained by introducing ANOVA-based gene selection (Fig. 11B) follows directly from the conceptual framework developed in the main text. ANOVA explicitly filters out genes whose expression does not vary across cell types within a tissue, thereby removing directions in gene space dominated by technical noise and dropout. This operation does not introduce new information; instead, it reshapes the representation so that remaining dimensions preferentially encode inter–cell-type variability. As a result, even simple linear decision boundaries become effective once the signal-to-noise ratio is sufficiently enhanced.

Figures 11C and D show that this improvement is consistent across tissues. Importantly, the relative ordering of tissues by difficulty is roughly preserved, indicating that ANOVA selection acts as a uniform denoising step rather than a tissue-specific optimization. Tissues with many closely related immune or stromal subtypes remain challenging, but their absolute performance improves substantially once irrelevant gene-level noise is removed.

A more granular view of this effect is shown in Fig. 12, which focuses on five representative tissues. When using scTOP alone (Fig. 12A), confusion between transcriptionally similar cell types is pervasive, particularly for rare or weakly differentiated subpopulations. After ANOVA selection (Fig. 12B), these same tissues exhibit sharply improved separation, despite the classifier and overall pipeline remaining entirely linear and nearly parameter-free.

Taken together, these results demonstrate that scTOP's limitations on Tabula Sapiens 2.0 do not arise from the use of linear methods, but from representing highly noisy data using overly coarse summary statistics. The ANOVA selection step directly addresses this issue by aligning the representation with the structure of the classification task. In a subsequent SI section, we analyze the noise properties of Tabula Sapiens 2.0 in detail and show explicitly how dropout and within–cell-type variability degrade centroid-based representations.

bioRxiv preprint doi: https://doi.org/10.64898/2026.02.11.705358; this version posted February 13, 2026. The copyright holder for this preprint (which was not certified by peer review) is the author/funder, who has granted bioRxiv a license to display the preprint in perpetuity. It is made available under aCC-BY 4.0 International license.

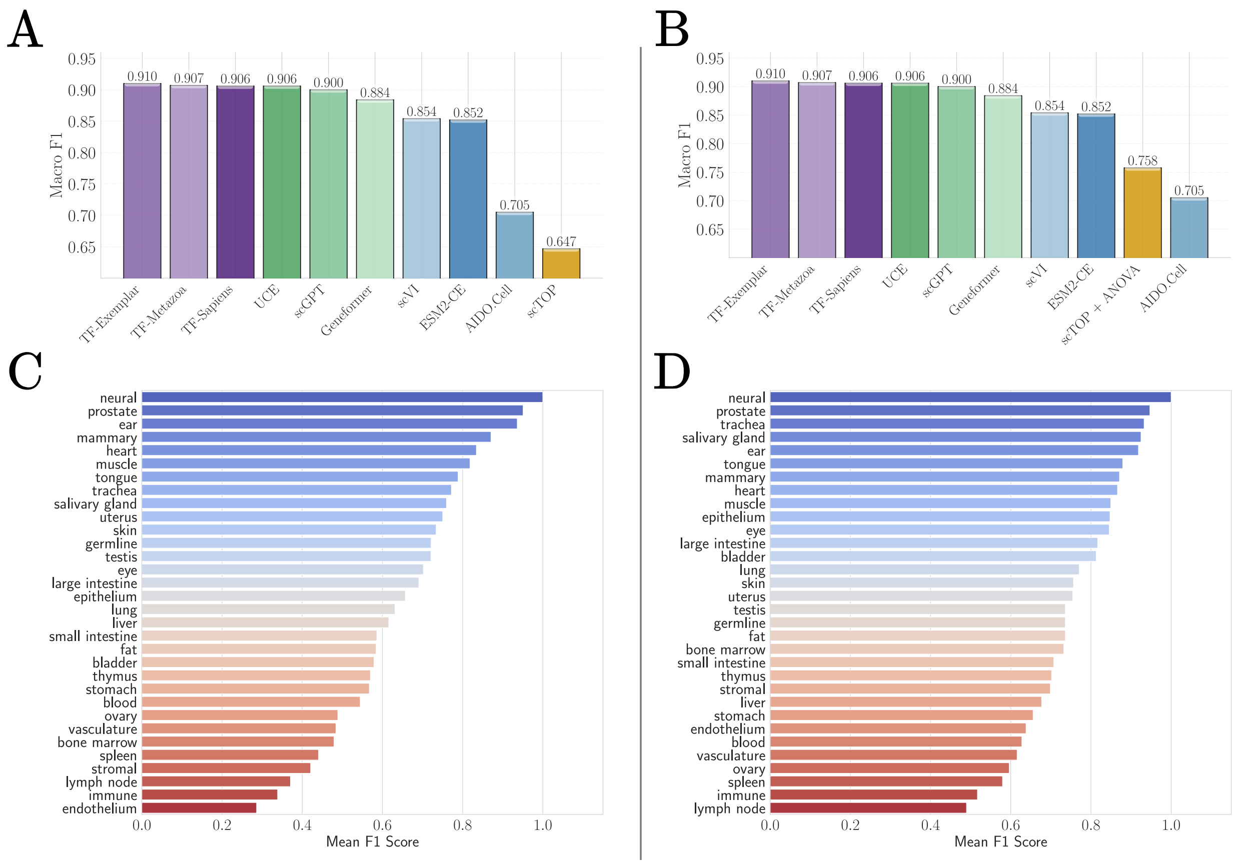

FIG. 11: **scTOP performance on Tabula Sapiens 2.0.** **A**: Macro F1-score of scTOP alone compared to foundation-model baselines. **B**: Macro F1-score after adding ANOVA-based gene selection. **C**: Per-tissue macro F1-scores using scTOP alone. **D**: Per-tissue macro F1-scores for scTOP with ANOVA selection.

3. Computational Cost, Throughput, and Scalability Analysis

Here we provide a detailed and quantitative comparison of inference-time computational requirements for transformer-based cellular foundation models and the scTOP-based pipelines used throughout this work. We compare these approaches along multiple independent axes: floating-point operations (FLOPs), throughput (cells processed per second), scalability with dataset size, energy consumption, carbon footprint, and monetary cost.

All comparisons are reported on a per-cell basis and are intentionally conservative, favoring foundation models whenever architectural details are not fully specified. Importantly, we focus exclusively on inference, which is the dominant operational regime for cell atlas annotation, cross-species trans-

bioRxiv preprint doi: https://doi.org/10.64898/2026.02.11.705358; this version posted February 13, 2026. The copyright holder for this preprint (which was not certified by peer review) is the author/funder, who has granted bioRxiv a license to display the preprint in perpetuity. It is made available under aCC-BY 4.0 International license.

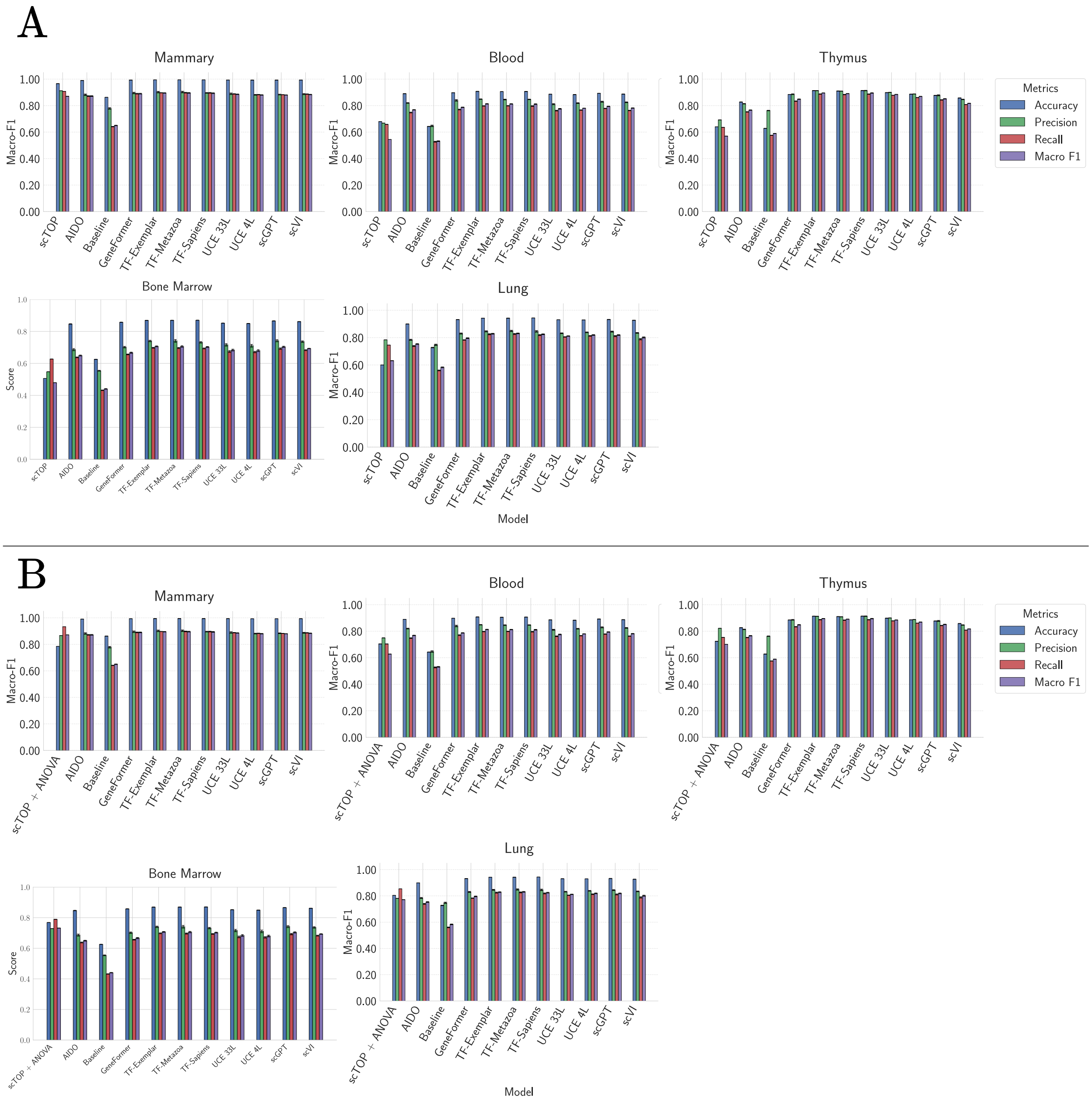

FIG. 12: **Detailed tissue-level performance. A**: scTOP alone across five representative tissues. **B**: scTOP with ANOVA-based gene selection on the same tissues.

fer, and large-scale biological analysis.

bioRxiv preprint doi: https://doi.org/10.64898/2026.02.11.705358; this version posted February 13, 2026. The copyright holder for this preprint (which was not certified by peer review) is the author/funder, who has granted bioRxiv a license to display the preprint in perpetuity. It is made available under aCC-BY 4.0 International license.

Algorithmic Decomposition of Inference Pipelines

a. scTOP. Inference with scTOP consists of three strictly defined steps: (i) elementwise preprocessing of gene expression values, (ii) projection of the processed expression vector onto a precomputed reference basis of cell-type order parameters, and (iii) selection of the maximal projection score. The reference basis is constructed once and reused across all target cells; its cost is amortized and therefore excluded from per-cell inference cost, consistent with standard benchmarking practice.

Let G denote the number of genes shared between reference and target datasets after ortholog mapping, and let K denote the number of reference cell types. The dominant operation is a dense matrix–vector multiplication of size $G \times K$, yielding an exact inference-time complexity of

$$\mathcal{O}(GK)$$

floating-point operations (FLOPs) per cell.

Across all experiments in this work, we observe $G \in [10^3, 5 \times 10^3]$ and $K \in [20, 150]$, corresponding to

$$2 \times 10^5 \text{ to } 6 \times 10^6 \text{ FLOPs per cell.}$$

b. TranscriptFormer. Inference with TranscriptFormer consists of a full forward pass through a large transformer-based encoder applied to each individual cell. As described in the original publication, TranscriptFormer models operate on gene tokens shared across species and compute contextualized cell embeddings via repeated blocks of multi-head self-attention and position-wise feedforward transformations. Inference therefore requires evaluating all transformer layers for every input cell, with no amortization across cells.

The TranscriptFormer family includes three released models of increasing scale. The largest model (*TF-Metazoa*) is trained on 112 million cells across twelve species and contains 444 million trainable parameters together with 633 million non-trainable parameters, for a total of over 1.07 billion parameters. Due to this scale, the processing of new cells for embedding generation effectively requires high-memory GPU hardware (e.g., A100-class accelerators).

Let n denote the number of gene tokens (up to a maximum sequence length of 2,047), $d = 2048$ the hidden dimension of the model, and $L = 12$ the number of transformer encoder layers. The dominant operations during inference arise from multi-head self-attention and dense linear projections. As for standard transformer architectures, the inference-time complexity per cell scales as

$$\mathcal{O}\big(L(n^2 d + nd^2)\big) ,$$

with the quadratic self-attention term $n^2 d$ dominating for biologically realistic values of n.

Using the explicit model configuration reported in the paper ($L = 12$, $d = 2048$), inference with TranscriptFormer necessarily requires on the order of

$$10^{11} \text{ to } 10^{12} \text{ floating-point operations per cell,}$$

bioRxiv preprint doi: https://doi.org/10.64898/2026.02.11.705358; this version posted February 13, 2026. The copyright holder for this preprint (which was not certified by peer review) is the author/funder, who has granted bioRxiv a license to display the preprint in perpetuity. It is made available under aCC-BY 4.0 International license.

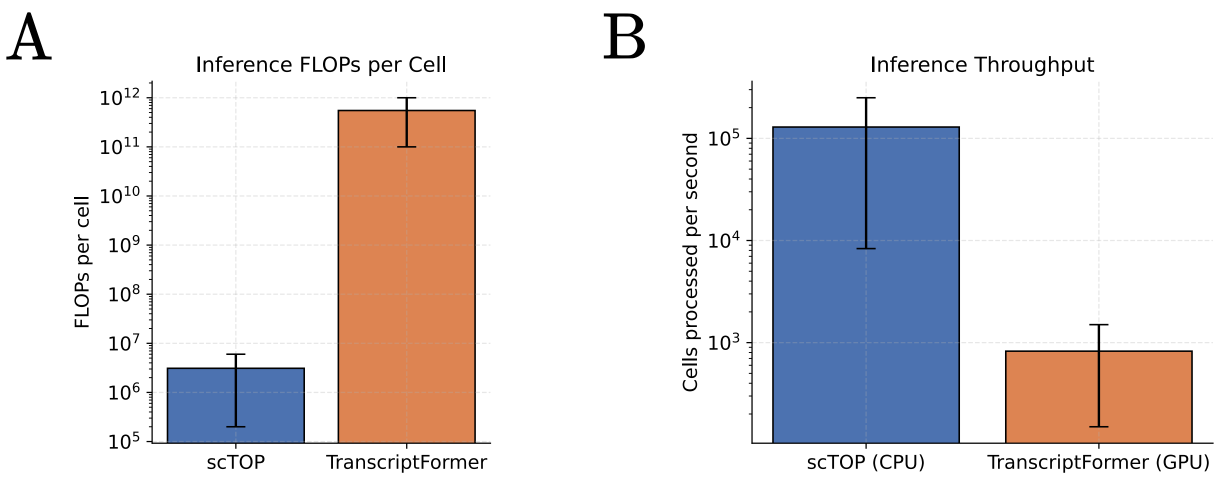

FIG. 13: Comparison between the cost for extracting embeddings using TranscriptFormer models and pre-processing data using scTOP. The error bars indicate that the results are estimates and depends on the size of the model used (such as TF-exemplar, TF-sapiens, and TF-Metazoa). **A**: Floating point operations per-cell comparison. scTOP pre-processing is 10^5 to 10^6 times cheaper than extracting embeddings with TranscriptFormer. **B**: scTOP is 10 to 10^3 faster in processing cells than TranscriptFormer. Notice however that scTOP uses CPU while TranscriptFormer uses GPU, *i.e.* is also much cheaper.

depending on the specific model variant and the number of genes included. This estimate is a lower bound and excludes additional overhead from memory access, embedding aggregation, and downstream classification.

As a consequence of this computational structure, TranscriptFormer inference throughput is limited to tens of cells per second even on modern GPUs. CPU-based inference, while theoretically possible, requires several seconds per cell due to the need to evaluate a billion-parameter transformer, resulting in throughput well below one cell per second and rendering CPU-based inference impractical for large-scale analyses.

Inference Throughput: Cells Processed per Second

To translate FLOP counts into throughput, we estimate sustained effective compute rates under realistic conditions. We assume 50 GFLOPs/s for CPU execution and 150 TFLOPs/s for GPU execution, corresponding to typical effective performance rather than peak theoretical throughput.

bioRxiv preprint doi: https://doi.org/10.64898/2026.02.11.705358; this version posted February 13, 2026. The copyright holder for this preprint (which was not certified by peer review) is the author/funder, who has granted bioRxiv a license to display the preprint in perpetuity. It is made available under aCC-BY 4.0 International license.

c. scTOP (CPU). Under these assumptions, scTOP achieves inference throughput of approximately

$$10^4\text{–}10^5 \text{ cells per second}$$

on commodity CPU hardware.

d. TranscriptFormer (GPU). On A100-class GPUs, TranscriptFormer achieves inference throughput on the order of

$$10^1\text{–}10^2 \text{ cells per second,}$$

depending on model variant and batch size.

e. TranscriptFormer (CPU). Running TranscriptFormer inference on CPU is technically possible but computationally impractical. Using the same assumptions, CPU-based inference yields throughput of at most

$$10^{-2}\text{–}10^{-1} \text{ cells per second,}$$

corresponding to several seconds per cell. As a result, TranscriptFormer inference on CPU is slower than scTOP by approximately

$$10^5\text{–}10^6 \text{ fold,}$$

and is not viable for atlas-scale analyses.

Scalability with Dataset Size

For a dataset containing N cells, total inference cost scales as

$$\text{FLOPs}_{\text{scTOP}} \sim \mathcal{O}(NGK), \qquad \text{FLOPs}_{\text{TF}} \sim \mathcal{O}(NLn^2d).$$

Because scTOP inference scales linearly with both dataset size and modest reference dimensionality, datasets containing millions of cells can be processed efficiently on commodity hardware. In contrast, TranscriptFormer inference scales superlinearly with gene count and requires specialized accelerators even for moderate dataset sizes.

At the scale of modern cell atlases ($N \sim 10^6$–10^7), total inference FLOPs differ by approximately

$$10^5\text{–}10^6 \text{ orders of magnitude.}$$

Energy Consumption and Carbon Footprint

To estimate energy usage, we adopt conservative efficiencies of 1.5 nJ/FLOP for CPU computation and 0.4 nJ/FLOP for GPU computation. Using a representative carbon intensity of 0.4 kg CO_2/kWh, we estimate per-cell emissions.

bioRxiv preprint doi: https://doi.org/10.64898/2026.02.11.705358; this version posted February 13, 2026. The copyright holder for this preprint (which was not certified by peer review) is the author/funder, who has granted bioRxiv a license to display the preprint in perpetuity. It is made available under aCC-BY 4.0 International license.

Under these assumptions, TranscriptFormer inference emits approximately

$$10^5–10^6$$

times more CO_2 per processed cell than scTOP. This estimate excludes the substantially larger carbon footprint associated with training large foundation models, which would further widen this gap.

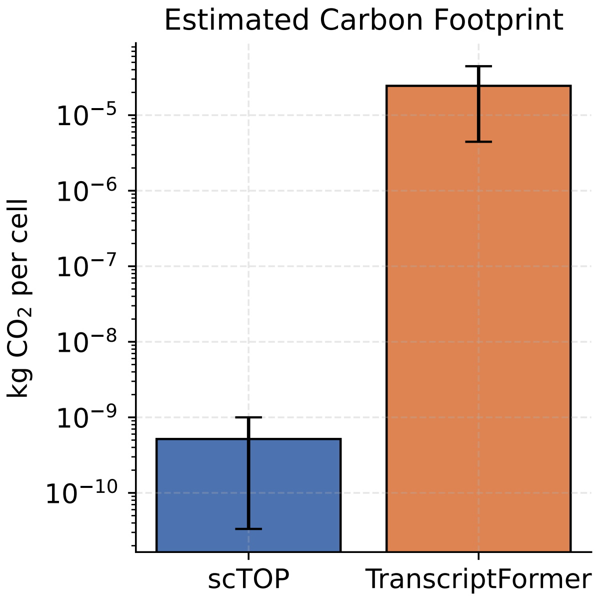

FIG. 14: CO_2 cost comparison between scTOP and TranscriptFormer during inference.

Monetary Cost of Inference

Assuming typical cloud pricing of $3/hour for CPU instances and $30/hour for A100-class GPUs, the per-cell monetary cost of TranscriptFormer inference exceeds that of scTOP by

$$10^4–10^5$$

orders of magnitude, depending on batch size and deployment configuration. This cost differential scales linearly with dataset size and rapidly dominates total analysis cost for atlas-scale studies.

bioRxiv preprint doi: https://doi.org/10.64898/2026.02.11.705358; this version posted February 13, 2026. The copyright holder for this preprint (which was not certified by peer review) is the author/funder, who has granted bioRxiv a license to display the preprint in perpetuity. It is made available under aCC-BY 4.0 International license.

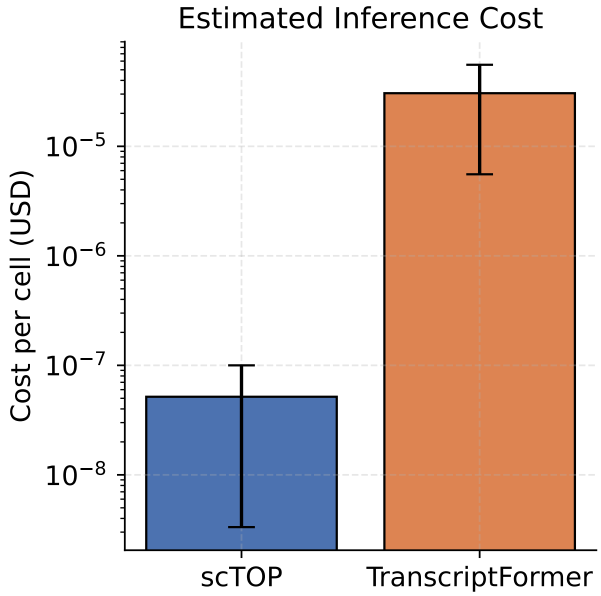

FIG. 15: Monetary comparison between scTOP and TranscriptFormer during inference.

Summary of Orders-of-Magnitude Differences

Across all metrics considered—inference-time FLOPs, throughput, scalability, energy consumption, carbon footprint, and monetary cost—scTOP outperforms TranscriptFormer by between

$$10^4 \text{ and } 10^6$$

orders of magnitude at inference time while achieving comparable or superior predictive performance on the same benchmarks.

These results demonstrate that the empirical success of large cellular foundation models cannot be interpreted independently of their extreme computational cost, and underscore the necessity of incorporating efficiency, scalability, and resource usage into benchmarking frameworks for biological representation learning.

4. Manifold analyses for more datasets

In this section we show the results of manifold analyses for more datasets beyond human spermatogenesis. We first show the results for other spermatogenesis datasets and then we show it for

bioRxiv preprint doi: https://doi.org/10.64898/2026.02.11.705358; this version posted February 13, 2026. The copyright holder for this preprint (which was not certified by peer review) is the author/funder, who has granted bioRxiv a license to display the preprint in perpetuity. It is made available under aCC-BY 4.0 International license.

four Tabula sapiens datasets chosen to represent different scores on our pipeline.

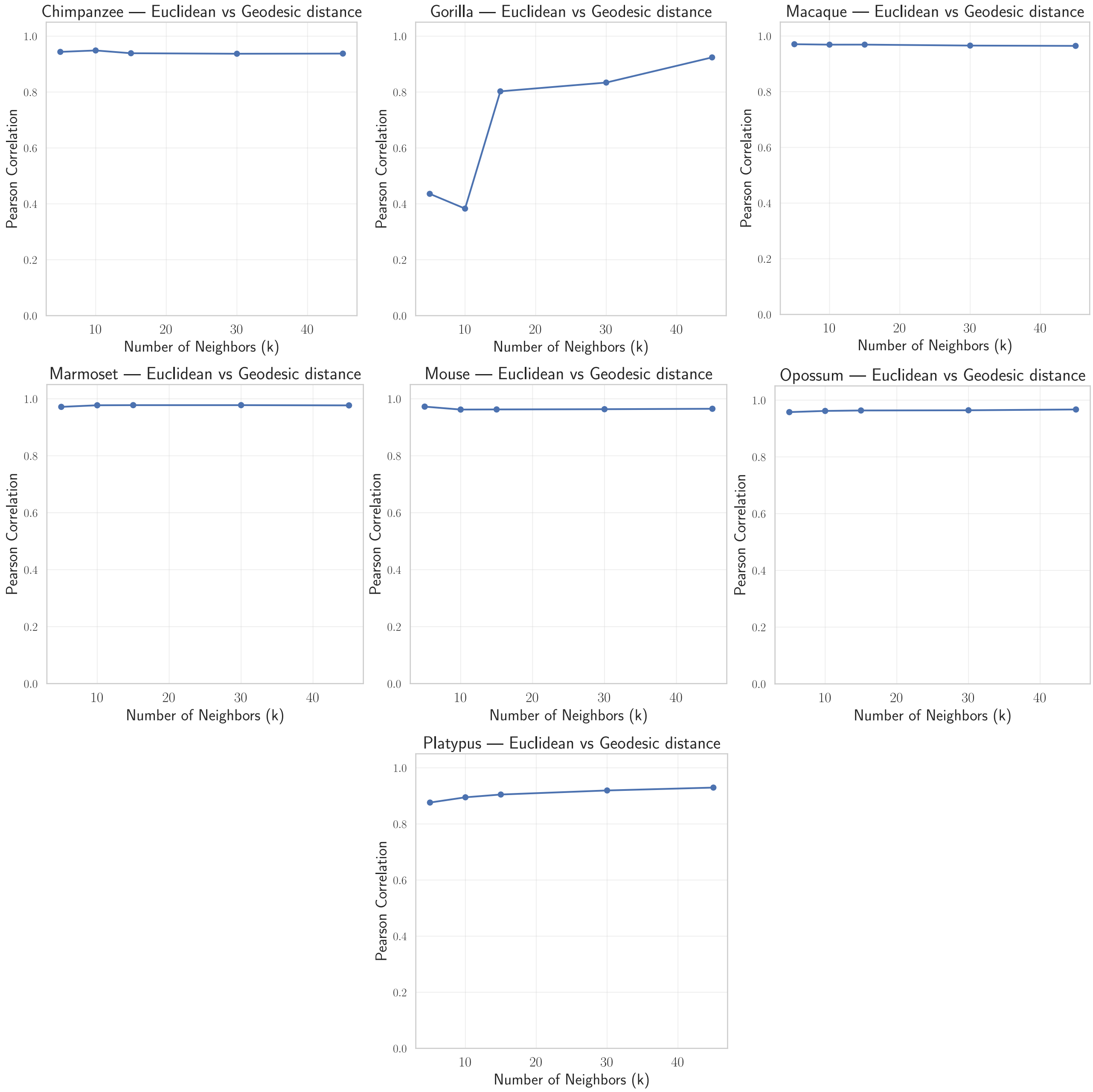

FIG. 16: Manifold analyses similar to the main text for the other seven spermatogenesis datasets.

bioRxiv preprint doi: https://doi.org/10.64898/2026.02.11.705358; this version posted February 13, 2026. The copyright holder for this preprint (which was not certified by peer review) is the author/funder, who has granted bioRxiv a license to display the preprint in perpetuity. It is made available under aCC-BY 4.0 International license.

In Fig. 16 we show the results of the same manifold analyses discussed in the main text but now for the other seven spermatogenesis dataset. We see that the results are similar to the human dataset, indicating that the near-linearity manifold feature remains true across datasets. The Gorilla dataset, on the other hand, yields unstable Isomap geodesics as a function of neighborhood size, suggesting that the kNN graph is not a robust manifold proxy for this dataset (e.g., disconnectedness at small k and/or short-circuit edges at larger k). Therefore the discrepancy reflects sensitivity of geodesic estimation to sampling/noise rather than a genuine change in tissue geometry.

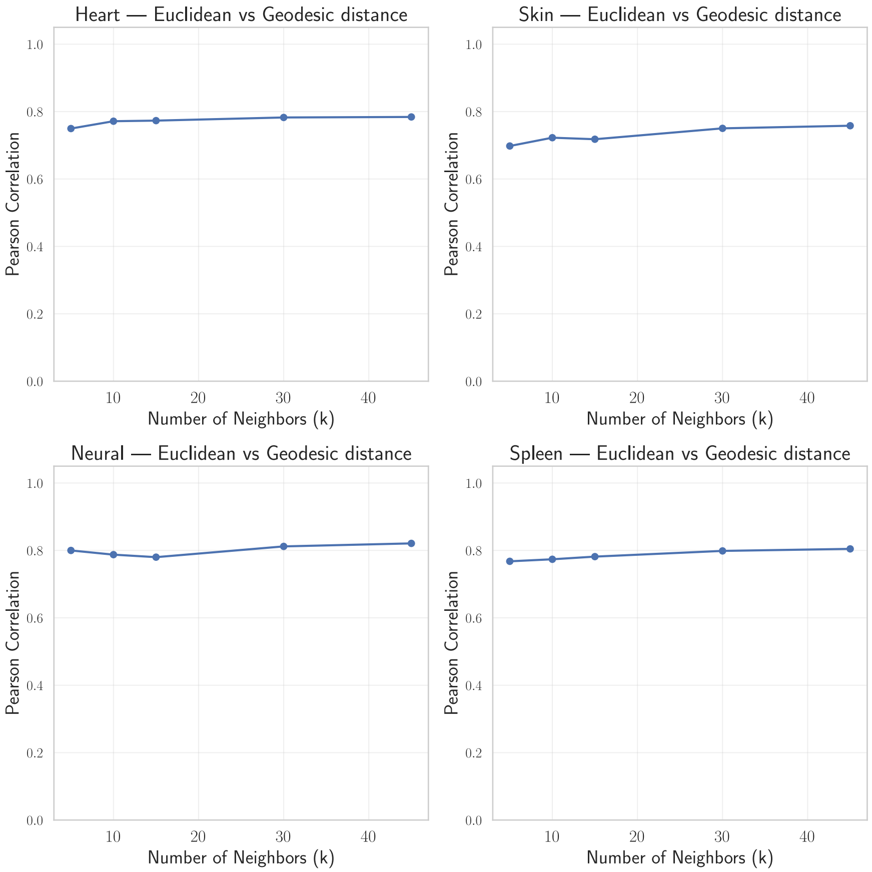

FIG. 17: Manifold analyses across four different tissues on Tabula Sapiens 2.0.

In Fig. 17 we see that after using the process of scTOP the four tissues displayed have a correlation of around 0.8 between Euclidean distances and geodesics computed using Isomap. We observe that even for spleen tissue, that is among the low scores tissues (see Fig. 4) we obtain this high correlation. We interpret this as an evidence that the scRNA-seq data lives in a space that is not highly non-linear or has folding properties or holes. Moreover, most of the curvature and complications in this data arises

bioRxiv preprint doi: https://doi.org/10.64898/2026.02.11.705358; this version posted February 13, 2026. The copyright holder for this preprint (which was not certified by peer review) is the author/funder, who has granted bioRxiv a license to display the preprint in perpetuity. It is made available under aCC-BY 4.0 International license.

from its technical noise and batch effects (see next section for discussions on this for toy datasets).

While Isomap provides a useful diagnostic for detecting non-linear structure by approximating geodesic distances on a neighborhood graph, its reliability depends critically on data quality and sampling density. In particular, Isomap assumes that local Euclidean neighborhoods faithfully approximate the underlying manifold; this assumption can break down in sparse, noisy, or heterogeneously sampled scRNA-seq datasets, where k-nearest-neighbor graphs may become disconnected or connected only through narrow bridges. In such regimes, estimated geodesic distances can become unstable with respect to the neighborhood size and may exaggerate apparent non-linearities that arise from technical noise, batch effects, or outliers rather than intrinsic biological structure. This sensitivity motivates using Isomap primarily as a qualitative probe of curvature, complemented by linear diagnostics and robustness checks as done throughout this work.

5. Understanding levels of noise in Tabula Sapiens

To further investigate the need of denoising in the Tabula sapiens dataset, as an illustrative example we provide a more detailed analysis of technical noise in cells from the heart tissue. While using scTOP to classify cell types (see Fig. 18A), we observed a surprising result: smooth muscle cells were mislabeled as distantly related cell types (erythrocyte, monocyte, and pericytes). While it is normal for cells that are biologically similar to get confused (e.g. atrial cardiac myocyte and ventricular cardiac muscle cell), misclassification involving cell types that are so developmentally dissimilar is rare. As can be see in Fig. 18B, the centroid of gene expression of smooth muscle cells in the training dataset is closer in gene space to, for example, cardiac endothelial cell and fibroblast of cardiac tissue than to erythrocyte and monocyte.

To understand this behavior, we plotted the PC1 and PC2 for smooth cells in the test set (see Fig. 19A). Even though these cells all share the same label, they form two clear and distinct clusters. Furthermore, one of these cluster that is largely classified correctly by scTOP and the other cluster is assigned to the wrong cell type (Fig. 19B). We noticed that these clusters differ in their technical noise. When we color the samples by the number of genes with at least one count in a cell, we see that the cluster of the left has a much higher dropout rate than the cluster on the right (see Fig. 19C). This pattern likely reflects technical artifacts arising from data collection and pre-processing rather than an intrinsic biology.

Fig. 19D shows a PCA of smooth muscle cells along with the cells for which they were confused (erythrocytes, monocytes, and pericytes). We notice that cells confused with pericyte indeed live closer to the pericyte cluster, cells confused with monocyte live closer to the monocyte cluster (see Fig. 19E). Meanwhile, cells labeled as erythrocyte are simply spread out over the whole PCA embeddings. Collectively, these observations suggest the source of scTOPs errors is likely technical noise.

These results also help explain why ANOVA selection helps in annotation tasks Since the differences in gene expression create two different clusters for a cell type, ANOVA acts as a denoiser of

bioRxiv preprint doi: https://doi.org/10.64898/2026.02.11.705358; this version posted February 13, 2026. The copyright holder for this preprint (which was not certified by peer review) is the author/funder, who has granted bioRxiv a license to display the preprint in perpetuity. It is made available under aCC-BY 4.0 International license.

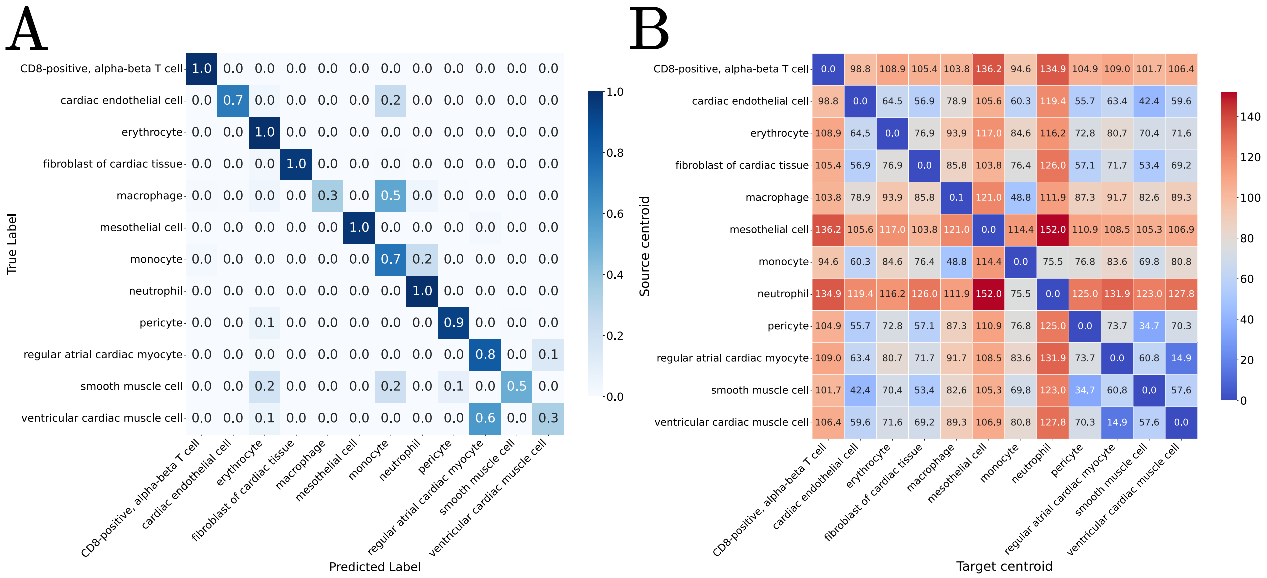

FIG. 18: **A**: Confusion matrix for heart tissue. **B**: Distance between centroids across cell types.

dimensions that create artificial differences between cells in the same cell type. Since it works by selecting genes that minimize variance within clusters and maximize variance between clusters, it effectively chooses genes that make each cell type cluster tighter while making it as further as possible to the others.

6. Linear Discriminant Analysis

In this section, we use this Linear Discriminant Analysis (LDA) to further probe the linear separability of cell types. After using the full pipeline described in Sec. IV, we use LDA to understand if this is already enough to find meaningful directions that separate different classes.

Unlike PCA, which is an unsupervised method that finds the axes of maximum total variance, LDA is a supervised method that finds the axes (linear discriminants) that best separate the known classes. It does this by maximizing the ratio of between-class variance to within-class variance. We apply LDA to the d-dimensional $\mathbf{z}_i$ vectors from the PCA step. For K classes, we first compute the class means $\mathbf{m}_k$ and the overall mean $\mathbf{m}$:

$$\mathbf{m}_k = \frac{1}{N_k} \sum_{i \in C_k} \mathbf{z}_i, \qquad \mathbf{m} = \frac{1}{N} \sum_{i=1}^{N} \mathbf{z}_i,$$

bioRxiv preprint doi: https://doi.org/10.64898/2026.02.11.705358; this version posted February 13, 2026. The copyright holder for this preprint (which was not certified by peer review) is the author/funder, who has granted bioRxiv a license to display the preprint in perpetuity. It is made available under aCC-BY 4.0 International license.

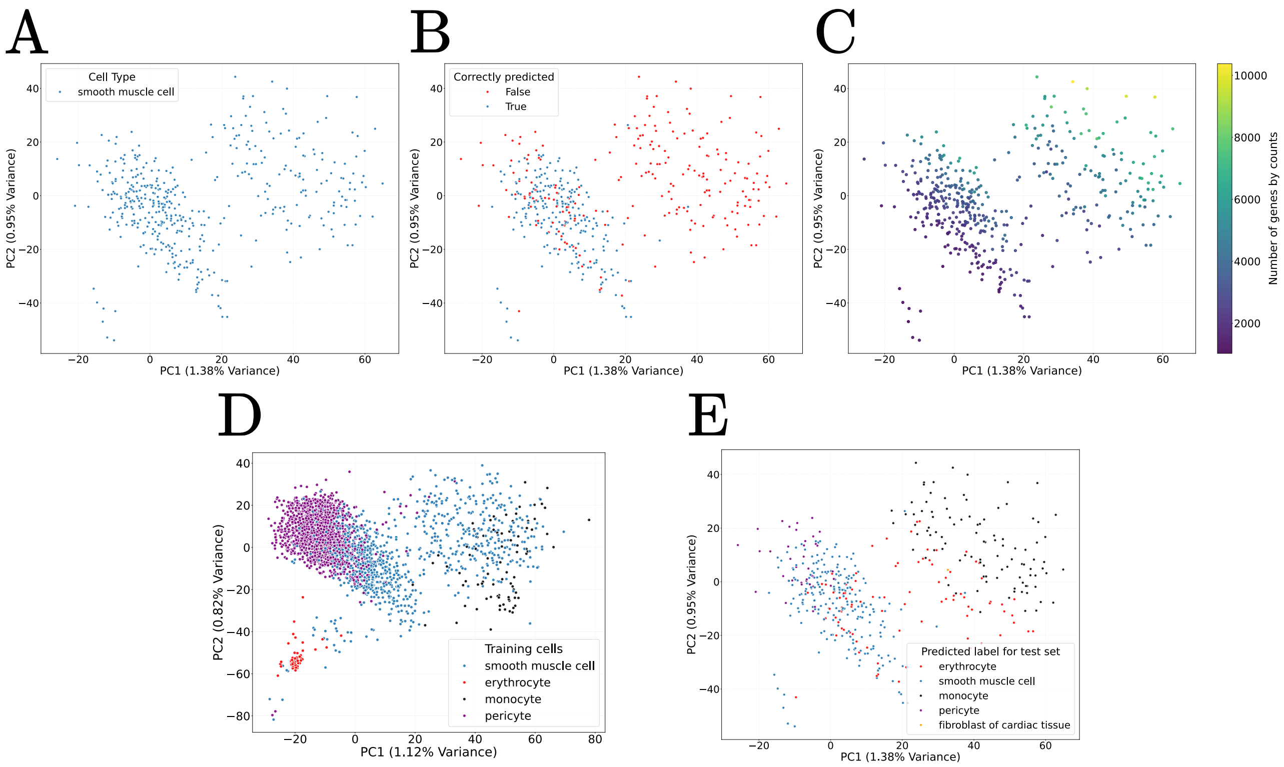

FIG. 19: **PCA study of smooth muscle cells**. **A**: Smooth muscle cells PC1 and PC2. **B**: PCA for smooth muscle cells colored by correct and incorrect annotation. **C**: Smooth muscle cells colored by number of genes with at least one count. **D**: Training samples PC1 and PC2 for smooth muscle cells and cell types that it gets confused to. **E**: Smooth muscle cells PC1 and PC2 colored by predicted label for it.

where C_k is the set of cells in class k and $N_k = |C_k|$. The within-class scatter matrix $\mathbf{S}_W$ and between-class scatter matrix $\mathbf{S}_B$ are:

$$\mathbf{S}_W = \sum_{k=1}^{K} \sum_{i \in C_k} (\mathbf{z}_i - \mathbf{m}_k)(\mathbf{z}_i - \mathbf{m}_k)^\top$$

$$\mathbf{S}_B = \sum_{k=1}^{K} N_k (\mathbf{m}_k - \mathbf{m})(\mathbf{m}_k - \mathbf{m})^\top$$

bioRxiv preprint doi: https://doi.org/10.64898/2026.02.11.705358; this version posted February 13, 2026. The copyright holder for this preprint (which was not certified by peer review) is the author/funder, who has granted bioRxiv a license to display the preprint in perpetuity. It is made available under aCC-BY 4.0 International license.

LDA finds the projection directions $\mathbf{w}_j$ by solving the generalized eigenvalue problem:

$$\mathbf{S}_B \mathbf{w}_j = \lambda_j \mathbf{S}_W \mathbf{w}_j.$$

The resulting projection vectors (eigenvectors) form a new basis $\mathbf{W}$ that projects the data into a new space of at most $K-1$ dimensions, where the classes are maximally separated.

We perform the same analyses as described in [27]. We train an LDA in donors from 1 to 16, then apply the trained model to donors 17-31. Importantly, this does not involve any deep learning. We first use this method to separate 5 cell types in spleen tissue (CD4+ alpha-beta T cell, CD8+ alpha-beta T cell, endothelial cell, monocyte, neutrophil, and plasma cell) as shown in Fig. 20A. We see that we are able to get similar qualitative results as in [27] using LDA. Moreover, we test if this pipeline can separate cells from a same cell type coming from different tissues. As shown in Fig. 20B, we are able to distinguish different tissues for monocytes as done in [27].

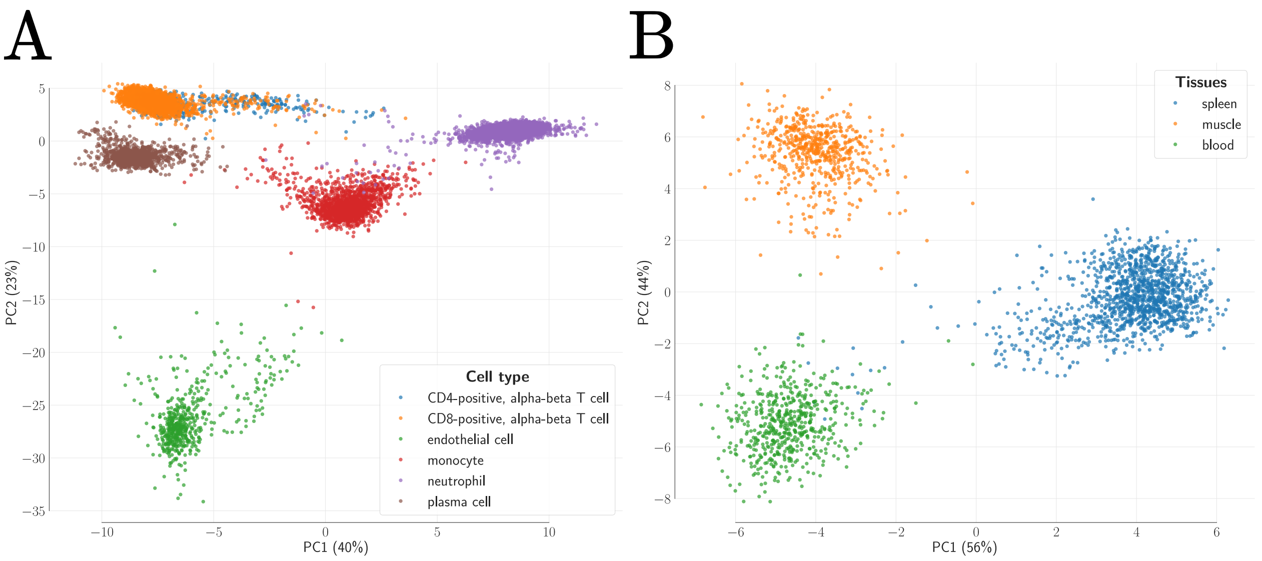

FIG. 20: **LDA analyses**. **A**: PC1 and PC2 projections of spleen tissue cells after using LDA show separability of different cell types after use of our pipeline. **B**: PC1 and PC2 of monocyte cells show separation across tissues after the use of LDA.
